# Electroacupuncture at ST-36 Protects Interstitial Cells of Cajal via Sustaining Heme Oxygenase-1 Positive M2 Macrophages in the Stomach of Diabetic Mice

**DOI:** 10.1155/2018/3987134

**Published:** 2018-04-26

**Authors:** Lugao Tian, Shuangning Song, Beibei Zhu, Shi Liu

**Affiliations:** Division of Gastroenterology, Union Hospital, Tongji Medical College, Huazhong University of Science and Tenchnology, Wuhan, China

## Abstract

**Background:**

Electroacupuncture (EA) at ST-36 has been reported to improve delayed gastric emptying and protect the networks of ICC in diabetic models. However, the mechanisms of the effects of EA are still unclear. The purpose of this study was to investigate whether the HO-1 positive M2 macrophages participate in the protective effects of EA for the ICC networks.

**Methods:**

Male C57BL/6 mice were randomized into five groups: the normal control group, diabetic group (DM), diabetic mice with sham EA group (SEA), diabetic mice with low frequency EA group (LEA), and diabetic mice with high frequency EA group (HEA). ICC network changes were detected by Ano1 immunostaining. F4/80 and HO-1 costaining was used to measure HO-1 positive macrophage expression. Western blot and PCR methods were applied to monitor HO-1, IL-10, and macrophage markers, respectively. The serum MDA levels were detected by a commercial kit.

**Results:**

This study presents the following results: (1) Compared with the control group, ICC networks were severely disrupted in the DM group, but no obvious changes were found in the LEA and HEA groups. (2) Many HO-1 positive macrophages could be observed in the LEA and HEA groups, and the expression of HO-1 was also markedly upregulated. (3) The IL-10 expression was obviously upregulated in the LEA and HEA groups. (4) The serum MDA levels were decreased in the real EA group. (5) When compared to the DM group, the expression of CD163 and Arg-1 was increased in the LEA and HEA groups, but the iNOS expression was decreased.

**Conclusion:**

The protective effects of EA on the networks of ICC may rely on the HO-1 positive macrophages to mediate anti-inflammatory and antioxidative stress effects.

## 1. Background

Gastroparesis, a more common complication of diabetes, is characterized as a delayed emptying of stomach contents and accompanied with no mechanical obstruction. The irregular delivery of contents and mismatched hormonal responses generated by gastroparesis can aggravate the difficulty of glycemic control and further increase the rate of hospitalization and mortality in diabetic patients. So far, although various pharmaceutical and nondrug treatments have been carried out, the curative effects of diabetic gastroparesis are as yet dissatisfactory.

Recently, electroacupuncture (EA), which is a combination of the use of traditional acupuncture and an electrical current, has shown a potential role in regulating gastric motility and treating gastroparesis. It is reported that EA was able to alleviate dyspeptic symptoms and promote solid gastric emptying in diabetic patients with gastroparesis [1]. Our previous study demonstrated that EA at ST-36 could also increase gastric motility and promote gastric emptying in diabetic rats [2]. However, the mechanisms of EA on the gastric motility are still unclear.

Over the past years, the interstitial cells of Cajal (ICC) of Auerbach's plexus (AP) in the stomach have been well established to generate pacemaker activity and propagate slow waves. Several studies have shown that diabetic gastroparesis was accompanied with disrupted ICC networks in the stomach [3, 4]. Thus, the injured ICC in diabetes is recognized as one of the main contributors for the delayed gastric emptying. In our previous study, we found that both low- and high-frequency EA could rescue the c-Kit positive ICC networks, which may be the main reason for the improvement in gastric motility [2]. But how EA protects ICC is not understood.

Lately, the upregulated heme oxygenase-1 (HO-1) in macrophages has been proposed as a crucially protective factor for ICC in diabetes. Loss of upregulation of HO-1 in the stomach was reported to result in increased reactive oxygen species, loss of ICC, and development of gastroparesis in diabetic animal models [5]. HO-1 is an inducible isoform of heme oxygenase that can catalyze heme degradation and be upregulated by various factors such as inflammatory cytokines and oxidative stress. Several studies have suggested that the protective role of HO-1 might rely on the anti-inflammatory and antioxidative stress effects [5, 6]. However, whether the effects of the anti-inflammatory and antioxidative stress of HO-1 are involved in the protective effects of EA on ICC in diabetes needs to be investigated.

At present, polarized macrophages have been classified in two main groups: classically (M1) activated macrophages with proinflammatory effects and alternatively (M2) activated macrophages with anti-inflammatory effects [7]. HO-1 is primarily expressed on resident M2 macrophages [6]. Under the condition of diabetes, up to now, to the best of our knowledge, no study has shown the effects of EA on the two types of macrophages.

So, the aims of this study are to investigate macrophage changes and the HO-1 positive M2 macrophage expression after being treated with EA and to further explore whether the antioxidative stress and anti-inflammatory effects mediated by the HO-1 positive M2 macrophages participate in the protective effects of EA on ICC in diabetic mice.

## 2. Materials and Methods

### 2.1. Experimental Animals

Six-week-old male C57BL/6 wild-type mice were used in this study. The mice were obtained from Beijing Hua Fukang Biotechnology Co. Ltd. (Beijing China). They were housed in stainless steel cages maintained on a 12-hour light-dark cycle with a temperature condition of 21–23°C and allowed to get food and sterile water ad libitum. Before the normal study started, the mice had been adapted to the laboratory circumstances for at least one week. All of the mice received human care and all experimental procedures were approved by the Animal Care and Use Committee of Tongji Medical College, Huazhong University of Science and Technology.

### 2.2. Diabetic Model

All the mice were fasted for 24 hours, but could drink water freely. Then, the method of using a single intraperitoneal injection of streptozotocin (STZ, Sigma-Aldrich, St. Louis, MO, USA) to induce type 1 diabetic mellitus was applied. STZ was prepared in 0.1 mol/l citrate buffer and applied at a dose of 150 mg/kg for each mouse. The mice in the control group were only injected the same dose of citrate buffer. A week later, the blood glucose level of each mouse was measured by collecting a drop of whole blood from a small skin incision at the tip of the tail. Once the blood glucose level exceeded 250 mg/dl, we considered the diabetic model successful.

### 2.3. Experimental Protocols

All the mice were randomly placed into five groups (12 mice/group), including the control group, diabetic group (DM), diabetic mice with sham EA group (SEA, only acupuncture, no electric current, 30 min/day), diabetic mice plus low frequency EA group (LEA, 10 Hz, 1–3 mA, 30 min/day), and diabetic mice plus high frequency EA group (HEA, 100 Hz, 1–3 mA, 30 min/day). EA was executed at 9:00–9:30 AM every day for eight weeks and all of the parameters applied in this study were selected according to our previous studies [2].

In this study, an electrical stimulator (G6805-2A; Shanghai Huayi Medical Instrument Co. Ltd., Shanghai, China) was applied in the EA groups. The acupoint ST-36 of a mouse was at the posterolateral knee of the bilateral hind limbs, at about 2 mm under the fibular head [8]. A pair of stainless steel needles (0.16 × 7 mm) were used to acupuncture the ST-36 at a depth of 2-3 mm. When the bilateral hind limbs were slightly trembling, the electric current was satisfactory. To remove the restraint stress, all the mice had been fastened in a cage 30 min/day for one to two weeks before EA started. After eight weeks of EA, all the mice were sacrificed and stomach specimens were used for immunological staining, Western blot, and RT-PCR.

### 2.4. Immunofluorescence Staining

The composition of Kreb's solution used in this study included (in mmol/l) 118.1 NaCl, 4.8 KCl, 25 NaHCO_3_, 1.0 NaH_2_PO_4_, 1.2 MgSO_4_, 11.1 glucose, and 2.5 CaCl_2_ with a pH of 7.3-7.4. The freshly removed stomach was placed into the oxygenated Kreb's solution, then cut off along the lesser curvature with the gastric contents clearly washed away. Next, the stomach tissue was stretched over the surface of a Sylgard-coated dish with the mucosa face up. After the mucosa of the stomach was peeled away by a pair of forceps, the whole-mount preparations were fixed in ice-cold acetone for 10 minutes. The preparations were washed by 1 × PBS for three times and each time sustained for 10 minutes. Nonspecific binding was blocked with 5% normal goat serum containing 0.3% Triton X-100 for 2 hours. Then, the preparations were incubated with primary antibodies diluted in primary antibody dilution buffer containing 0.3% Triton X-100 for 24 hours at 4°C. These primary antibodies included rat anti-F4/80 (1 : 100, Abcam, Cambridge, MA, USA), rabbit anti-Ano1 (1 : 200, Abcam, Cambridge, MA, USA) and rabbit anti-HO-1 (1 : 200, Abcam, Cambridge, MA, USA). A single primary antibody or two primary antibodies together were preformed. After washing with 1 ×PBS, Dylight 488, goat anti-rat (Abbkine) and Dylight 549, goat anti-rabbit (Abbkine) were used to detect the specific labeling. Both the secondary antibodies were diluted in 1 × PBS containing 0.5% Triton X-100 and incubated for 2 hours. The solution without primary antibodies served as the negative controls. Preparations were examined by a confocal microscope (Olympus, Tokyo, Japan).

### 2.5. Western Blot Analysis

The fresh-frozen tissues of the stomach were homogenized by RIPA buffer with a protease inhibitor and the homogenates were centrifuged at 12,000 rpm for 16 min at 4°C. Then, all the supernatants were gathered as the protein. The bicinchoninic acid (BCA) method was used to evaluate the concentration of protein. Eighty micrograms of protein were separated by 10% sodium dodecyl sulfate-polyacrylamide gel electrophoresis (SDS-PAGE) and then transferred to a PVDF membrane. Nonspecific binding sites were blocked by 5% nonfat dry milk. Later, these membranes reacted with rabbit anti-HO-1 (1 : 5000; Abcam, Cambridge, MA, USA), rat anti-IL-10 (1 : 500; R&D Systems, Minneapolis, USA), and rabbit anti-mouse GAPDH (1 : 2000; GeneTex Inc., San Antonio, TX, USA) primary antibodies overnight at 4°C. After the membranes were incubated with an HRP-linked secondary antibody (HRP-linked goat anti-rat or HRP-linked goat anti-rabbit, 1 : 2000) for 60 min at room temperature, the bands were detected by a chemical reaction with an enhanced chemiluminescent agent (ECL; Thermo Fisher Scientific Inc., USA). The blot was subjected to autoradiography and Quantity One (Bio-Rad Technical Service Department, Version 4.6.2) was used to evaluate the band intensity.

### 2.6. mRNA Expression Analysis

The mRNA expression levels were detected by real-time quantitative reverse transcription PCR (qPCR). Total RNA of stomach tissues was extracted with a TRIzol reagent. After that, RNA was converted into cDNA using PrimeScript RT Master Mix (Takara, Otsu, Japan) on the basis of the manufacturer's instructions. The specific primer sequences used in this study are listed in Table 1. The process of qPCR was implemented as described by the manufacturer using SYBR Premix Ex Taq II (Takara) and Roche LightCycler® 480 (Roche, Switzerland) was used for detection. The transcript levels of each target gene were normalized by glyceraldehyde-3-phosphate dehydrogenase (GAPDH), and the relative expression levels were calculated according to the power formula: 2^−*∆*CT^(*∆*CT = CT_Target_ − CT_GAPDH_).

### 2.7. Serum Malondialdehyde (MDA) Level

A commercial MDA detection kit (Nanjing Jiancheng Bioengineering Institute, Nanjing, China) was used to detect the serum MDA level according to the instruction of the kit. 200 *μ*l of serum of each sample was used to detect the MDA level. The absorbance of the samples was recorded at 560 nm by a spectrophotometer (U-2900, Hitachi, Japan) and then used to calculate the serum MDA concentration in accordance with the formula that was provided in the instructions.

### 2.8. Statistical Analysis

All data were presented as means ± SEM and one-way ANOVA was employed to compare the difference among multiple groups. *P* < 0.05 was regarded as statistically significant difference. Statistical analysis was carried out with SPSS 17.0 (SPSS Inc., Chicago, IL).

## 3. Results

### 3.1. Effects of EA on ICC

**Figure 1:**
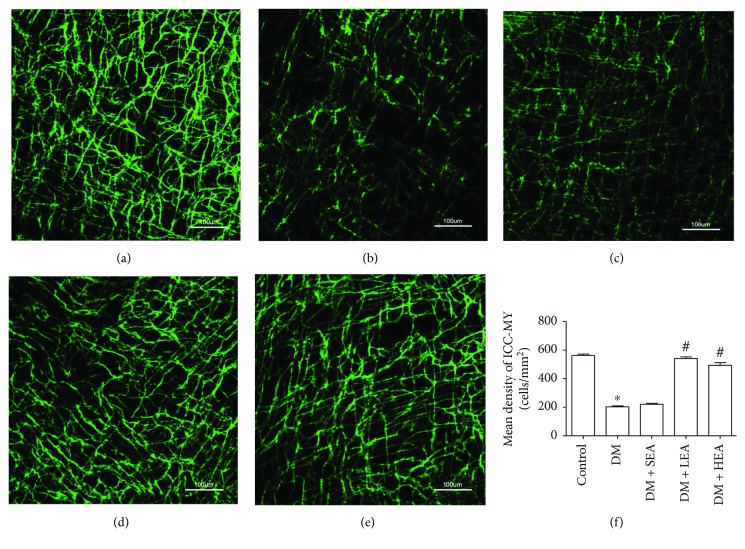
Immunofluorescence of ICC networks labeled in Auerbach's plexus by Ano1. Plenty of ICC with long and abundant branches were observed in the control group (a). ICC networks were heavily decreased with disrupted processes in the DM group and the SEA group (b, c). However, in the LEA and HEA groups, the networks of ICC presented near the normal levels in the control group (d, e). Quantitative analysis of ICC density was performed in each group (f). *N* = 5 for each group, and two random fields (×200 magnification, 0.2607 mm^2^) per whole-mount preparation were used. ^∗^*P* < 0.05 compared with the control group, and ^#^*P* < 0.05 compared with the DM group. Scale bars = 100 *μ*m.

Changes of ICC networks in AP were revealed in Figure 1. In the control group, multipolar ICC with numerous branches were formed into an intact cellular network (Figure 1(a)). However, in the DM group, Ano1 positive cells appeared with slender cell bodies and disrupted processes (Figure 1(b)), and the density of ICC was obviously decreased compared with the control group (*P* < 0.001) (Figure 1(f)). The density of ICC in the SEA group was not significantly altered compared to the DM group (*P* = 0.283) (Figures 1(c) and 1(f)). Nevertheless, an almost intact cellular network was found in the LEA and HEA groups (Figures 1(d) and 1(e)). When compared with the DM group, the ICC density was increased in the LEA and HEA groups (both *P* < 0.001) (Figure 1(f)). However, there were no obvious differences between the LEA group and the HEA group (*P* = 0.302) for the density of ICC networks.

### 3.2. Effects of EA on HO-1 Expression

**Figure 2:**
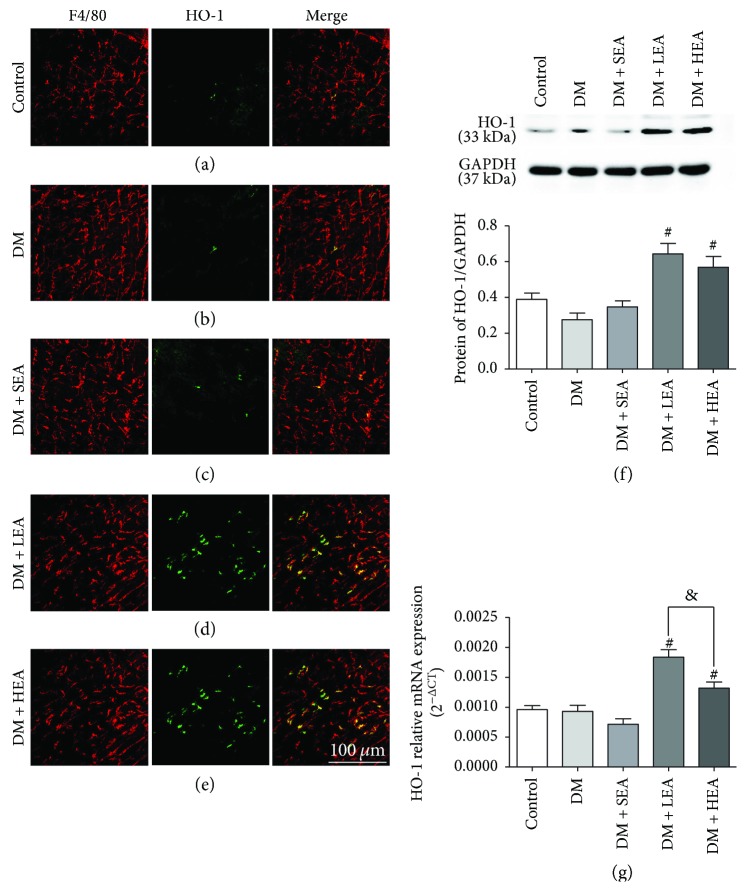
Immunofluorescence of HO-1 positive macrophages labeled by F4/80 and HO-1 and expression of HO-1 protein and mRNA in the stomach. (a–e) The costaining of HO-1 and F4/80 at the level of Auerbach's plexus for each group is shown. There were nearly no HO-1 positive cells in the control group, the DM group, and the SEA group, but many HO-1 positive macrophages were observed in the LEA and HEA groups. (f–g) The HO-1 protein expression and mRNA expression in the stomach is represented. Scale bars = 100 *μ*m and refers to all panels. *N* = 4 or 6 for each group. ^#^*P* < 0.05 compared with the DM group, and ^&^*P* < 0.05 among the LEA group and the HEA group.

F4/80 and HO-1 were colocalized to detected HO-1 macrophage expression in AP (Figure 2). Only a very few HO-1 positive and F4/80 positive cells were observed in the control, DM, and SEA groups (Figures 2(a)–2(c)), but abundant HO-1 positive and F4/80 positive cells could be found in the LEA and HEA groups (Figures 2(d)–2(e)).

To further evaluate HO-1 expression, Western blot and PCR techniques were used. Compared with the control group, the HO-1 protein expression levels had no significant difference in the DM group (*P* = 0.452) and the SEA group (*P* = 0.958) (Figure 2(f)). Compared to the DM group, the HO-1 protein expression was also significantly elevated in the LEA group (*P* = 0.001) and the HEA group (*P* = 0.007). No difference was found between the LEA group and the HEA group for the HO-1 protein expression (*P* = 0.978). The same tendency was observed for the HO-1 mRNA expression, but there was an obvious difference between the LEA group and the HEA group (*P* = 0.001) (Figure 2(g)).

### 3.3. Effects of EA on IL-10 Expression

**Figure 3:**
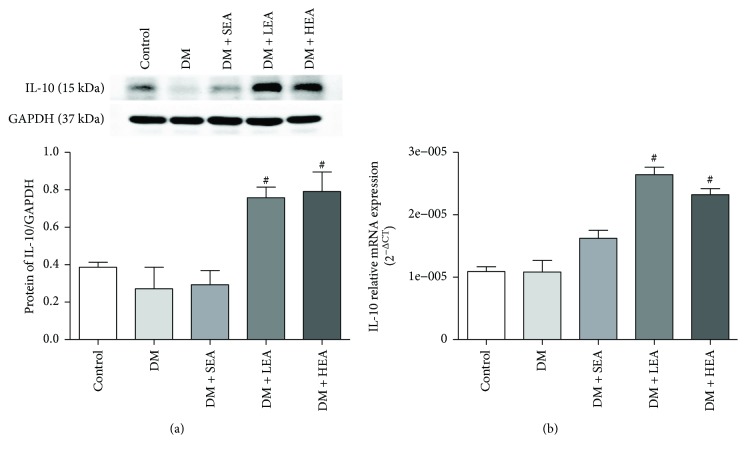
Expression of IL-10 protein and mRNA in the stomach. Compared with the control group, the expression of IL-10 protein was significantly increased in the LEA and HEA groups, but no obvious changes in the DM and SEA groups (a). The IL-10 mRNA expression had similar changes in each group (b). *N* = 5 or 6 for each group. ^#^*P* < 0.05 compared with the DM group.

Western blot analysis and PCR methods were applied to detect the IL-10 expression in the stomach (Figure 3). Compared to the control group, there were no significant differences for the protein expression of IL-10 in the DM group (*P* = 0.310) and the SEA group (*P* = 0.965) (Figure 3(a)). Compared with the DM group, the protein expression levels of IL-10 were also obviously upregulated in the LEA group (*P* < 0.001) and the HEA group (*P* < 0.001). However, there were no marked differences among the LEA group and the HEA group (*P* = 0.780) for the protein expression of IL-10. Similarly, when compared to the control group, there were also no differences for the mRNA of IL-10 in the DM group (*P* = 0.971) and the SEA group (*P* = 0.054). But when compared with the DM group, the mRNA of IL-10 were also markedly upregulated in the LEA group (*P* < 0.001) and the HEA group (*P* < 0.001). Also, no obvious difference was found between the LEA group and the HEA group for the expression of IL-10 mRNA (*P* = 0.085).

### 3.4. Effects of EA on the Level of Serum Malondialdehyde (MDA)

**Figure 4:**
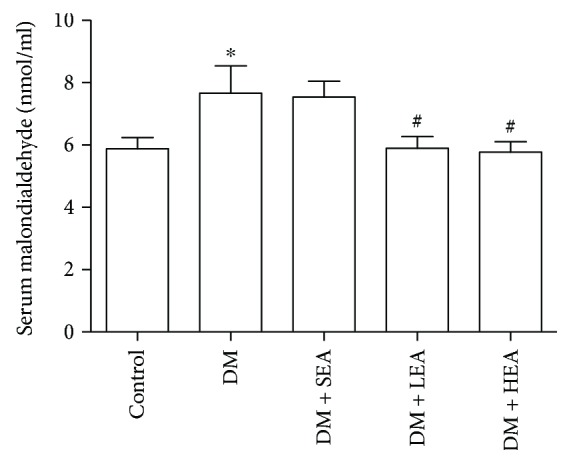
The serum malondialdehyde levels of each group. Compared to the control group, the serum malondialdehyde levels in the DM and SEA groups were elevated. However, compared with the DM group, in the LEA and HEA groups, the serum malondialdehyde levels were decreased to near the normal levels. *N* = 8 for each group. ^∗^*P* < 0.05 compared with the control group, and ^#^*P* < 0.05 compared with the DM group.

A commercial kit (Nanjing Jiancheng Bioengineering Institute, Nanjing, China) was used to measure the level of serum MDA (Figure 4). Compared with the control group, the serum MDA level was increased in the DM group (7.66 ± 0.87 versus 5.88 ± 0.35, *P* = 0.028) and the SEA group (7.54 ± 0.50 versus 5.88 ± 0.35, *P* = 0.040). However, by comparison with the DM group, the serum MDA level was decreased significantly in the LEA group (5.89 ± 0.37 versus 7.66 ± 0.87, *P* = 0.024) and the HEA group (5.77 ± 0.93 versus 7.66 ± 0.87, *P* = 0.016). Likewise, no obvious difference was found between the LEA group and the HEA group (5.98 ± 0.37 versus 5.77 ± 0.33, *P* = 0.869).

### 3.5. Effects of EA on the mRNA Expression of Macrophage Markers

**Figure 5:**
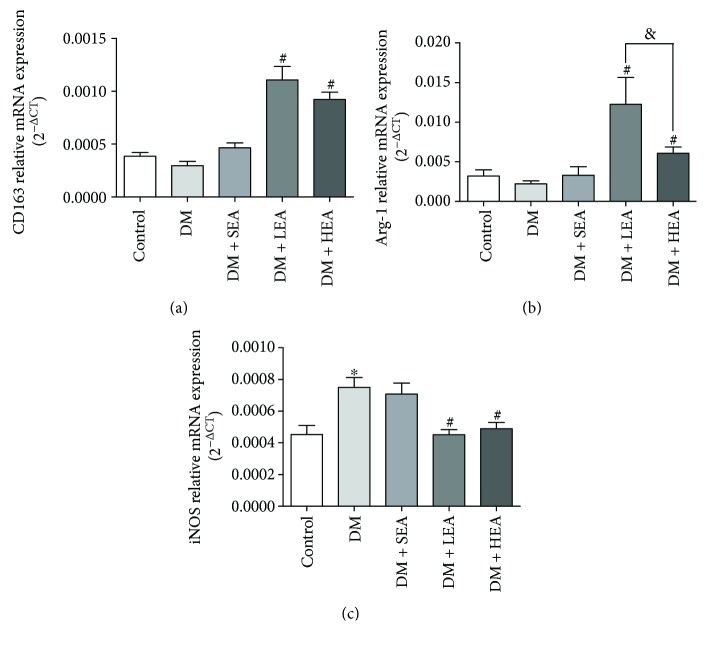
Expression levels of CD163, arginase-1, and iNOS mRNA in each group: (a) changes of mRNA expression levels of CD163, (b) arginase-1, and (c) iNOS in each group, respectively. *N* = 5 or 6 for each group. ^∗^*P* < 0.05 compared with the control group, ^#^*P* < 0.05 compared with the DM group, and ^&^*P* < 0.05 among the LEA group and the HEA group.

The PCR method was used to detect a part of the classical macrophage markers in stomach tissues (Figure 5). Compared with the control group, there were no obvious difference in the DM group for the CD163 and arginase-1 (Arg-1) mRNA expression (*P* = 0.390 and *P* = 0.687, resp.), but inducible nitric oxide synthase (iNOS) mRNA expression was obviously increased in the DM group (*P* = 0.001). Nevertheless, compared with the DM group, in the LEA group and the HEA group, CD163 and Arg-1 mRNA expression was increased obviously (all *P* < 0.01), but iNOS mRNA expression was decreased significantly (*P* = 0.001 and *P* = 0.003, resp.). Among the LEA group and the HEA group, no difference was found for the CD163 and iNOS expression (*P* = 0.086, *P* = 0.619, resp.), but obviously increased Arg-1 expression was observed in the LEA group (*P* = 0.017).

## 4. Discussion

In the present study, we have revealed the changes of the networks of ICC and the HO-1 positive macrophages in the stomach after being treated with EA. The increased HO-1 positive M2 macrophages may be presented with a protective role of EA on ICC networks via the upregulated IL-10 and decreased MDA in diabetic mice.

Electroacupuncture as an optimized Chinese traditional medicine has been used to treat gastrointestinal disorders for many years. Several studies revealed that EA at ST-36 had shown accelerative effects on gastric motility [2, 9]. However, only a few studies were focused on the mechanisms of EA on gastric motility. As mentioned above, ICC as the origin of pacemaker activity and slow waves perform a critical role in regulating gastric motility. Although there are three subtypes of ICC that include ICC-AP, intramuscular ICC (ICC-IM), and submucosal ICC (ICC-SM) in the stomach tissues, only the ICC-AP has been verified to generate pacemaker activity and slow waves [10]. Moreover, in the spatial structure, ICC-AP has also been found to have a closer relationship to the resident macrophages in muscularis [11]. It has been proposed that EA might help to reverse the pathological changes of ICC in diabetic rats [12]. Our previous study found the accelerative effects of gastric emptying by low- and high-frequency EA in diabetic rats; these may be attributed to the nearly unbroken ICC networks [2]. In the current study, we showed the variations of ICC networks stained by Ano1 after being treated with EA which were similar to the changes of ICC networks stained by c-Kit in a previous study. No nonspecific staining was found in this study, suggesting that Ano1 is a more reliable marker to monitor the ICC changes in diabetes and EA models. So, the unspoilt ICC networks labeled with Ano1 by low- and high-frequency EA also demonstrated the protective effects of EA on ICC networks. Nevertheless, reports about the mechanisms of the protective effects of EA on ICC are very scarce.

In recent years, HO-1 has been found to protect the ICC networks and defend against gastroparesis. In NOD mice, the HO-1 positive macrophages that disappeared in the stomach could result in c-Kit positive ICC loss and delayed gastric emptying [5]. Mogami et al. also reported that the impaired upregulation of the HO-1 expression might lead to damaged ICC in the gastric antrum in STZ-induced diabetic rats [13]. All of the changes could be reversed simultaneously by the induction of HO-1 expression in these diabetic animal models [5, 13]. Unfortunately, in diabetic patients with gastroparesis, the inducer of HO-1 could not sustain the increased HO-1 levels for a long time and failed to improve gastroparesis [14]. These reports may hint that HO-1 is very important to sustain ICC survival and protect gastric function in diabetes. So far, although EA has shown a protective role on ICC networks, no study about HO-1 has been applied to investigate the protective effects of EA on ICC. To our best knowledge, we firstly found that both low- and high-frequency EA could promote the HO-1 positive macrophage expression. Thus, the persistently expressed HO-1 positive macrophages may be the possible reason why EA protect the networks of ICC in diabetic mice.

The protective property of HO-1 on ICC is believed to be due to the effect of anti-inflammation. A deficiency of HO-1 in human and animal models could result in chronic inflammation [15]. IL-10 is one of the products of HO-1 positive macrophages, which has been well characterized with a powerful anti-inflammatory property. Studies have indicated that the anti-inflammatory effect of IL-10 might be achieved by reducing the antigen-presenting capacity and inhibiting cytokine synthesis [16]. IL-10 knockout mice were revealed to have severe inflammation and damaged ICC networks [17]. Meanwhile, IL-10 is also a potent inducer for the expression of HO-1 in macrophages, which may further enhance the anti-inflammatory effects of HO-1 [18]. Recently, Choi et al. found that IL-10 might increase gastric emptying via upregulating HO-1 and repairing ICC networks in diabetic mice [19]. Our present study also demonstrated that low- and high-frequency EA could increase the IL-10 expression levels, suggesting that the protective effects of EA on ICC may rely on the anti-inflammatory effect mediated by HO-1 positive macrophages.

The protective property of HO-1 on ICC has also been proposed owing to the effect of antioxidative stress. HO-1 deficient mice were reportedly accompanied with increased oxidative stress [20]. The increased oxidative stress in diabetes may lead to excessive lipid peroxidation and cellular injury, which can be reflected by serum malondialdehyde (MDA) levels [5]. Kaji et al. found that oxidative stress could impair pacemaker function of murine interstitial cells of Cajal [21]. Choi et al. further showed that HO-1 could protect ICC and reverse delayed gastric emptying by attenuating increased oxidative stress in diabetic mice [5]. In the current study, we also demonstrated that low- and high-frequency EA could reduce the serum MDA levels in STZ-induced diabetic mice, suggesting that the antioxidative stress effect of HO-1 positive macrophages may be involved in the protective effects of EA on ICC.

According to the characteristics of the M1 and M2 macrophages, there is no doubt that the HO-1 positive macrophages belong to the M2 macrophages. Up to now, a few macrophage markers have been identified to distinguish the M1 and M2 macrophages. CD163 is the heme regulatory molecule that was reported to be strongly upregulated by IL-10 and preferentially expressed on M2 macrophages [22]. Arg-1 as another M2 macrophage marker competes L-arginine with iNOS, a marker of M1 macrophages [23]. The balance of Arg-1 and iNOS was suggested to reflect the balance of M1 and M2 macrophages [24]. Aguiar et al. reported that EA at ST36 with low frequency has the capacity of generating M2 macrophages in a *Leishmania* infected model [25]. But few studies investigating macrophage changes can be found in the diabetic model treated with EA. Our present study revealed the increased expression of Arg-1 and CD163 coupled with a decreased expression of iNOS after being treated with EA, suggesting that both low- and high-frequency EA could promote the M2 macrophage expression and reduce the M1 macrophage expression in diabetic mice.

In summary, EA at ST-36 with low- and high-frequency can upregulate the M2 macrophages and decrease the M1 macrophage expression in the stomach of diabetic mice. After being treated with EA, the HO-1 and IL-10 expression levels were increased and the serum MDA levels were decreased. So, the protective effects of EA for the networks of ICC in diabetes may depend on the anti-inflammatory and antioxidative stress effects which were mediated by the HO-1 positive M2 macrophages.

## Figures and Tables

**Table 1:** List of designed primer sets for RT-PCR.

Gene	Primer	5′ → 3′	Size (bp)	Gene bank accession no.
GAPDH	Sense	TTCACCACCATGGAGAAGGC	237	XM-017592435.1
	Antisense	GGCATGGACTGTGGTCATGA		
HO-1	Sense	GTGACAGAAGAGGCTAAGACCG	241	NM-010442
	Antisense	ACAGGAAGCTGAGAGTGAGGAC		
IL-10	Sense	TGGACAACATACTGCTAACCGAC	111	NM-010548.2
	Antisense	CCTGGGGCATCACTTCTACC		
Arg-1	Sense	ATCAACACTCCCCTGACAACCA	255	NM-007482.3
	Antisense	TTCCATCACCTTGCCAATCC		
CD163	Sense	GTGGACTCTGAAGCGACGACA	102	NM-001170395.1
	Antisense	TCCGCCTTTGAATCCATCTC		
iNOS	Sense	CGGAGCCTTTAGACCTCAACAGA	243	NM-010927
	Antisense	TAGGACAATCCACAACTCGCTCC		
